# Successful Management of a Cesarean Scar Defect with Dehiscence of the Uterine Incision by Using Wound Lavage

**DOI:** 10.1155/2014/421014

**Published:** 2014-11-06

**Authors:** Akinori Ida, Yoko Kubota, Maiko Nosaka, Koichi Ito, Hiroshi Kato, Yoshiyuki Tsuji

**Affiliations:** Department of Obstetrics and Gynecology, Kobe Adventist Hospital, 4-1 Arinodai, 8-Chome, Kita-ku, Kobe 651-1321, Japan

## Abstract

Cesarean scar defects (CSDs) that can be visualized using transvaginal ultrasonography (TVUS) may cause prolonged menstruation, irregular genital bleeding, and secondary infertility; surgical repair is sometimes necessary. We present a case of CSD, with dehiscence of the uterine incision, which was managed using wound lavage. A 38-year-old woman (gravida 4, para 4) had delivered her third and fourth children by cesarean section. Upon the resumption of menstruation, 9 months after her second cesarean section, she demonstrated prolonged menstruation and the presence of a menstrual fistula due to dehiscence of the cesarean section incision from the myometrium to the serosa. We treated the defect by lavaging with a physiological saline solution. After lavaging the wound 3 times, spontaneous healing of the dehiscent muscle layer was successfully achieved. The treatment was complication-free and the healing of the muscle layer has been maintained for more than 8 months.

## 1. Introduction

A history of cesarean section is associated with an increased risk of cesarean scar pregnancy, uterine rupture, placenta previa, and placenta accreta during subsequent pregnancies. In 1995, Morris demonstrated the existence of a scar at the site of a cesarean section incision and reported the pathological changes associated with the scar site [1]. The study showed that the scar could be visualized using transvaginal ultrasonography (TVUS) as a cesarean scar defect (CSD). In addition, CSDs were shown to cause prolonged menstruation, irregular genital bleeding, and secondary infertility.

Here, we report a patient with CSD who demonstrated dehiscence of the cesarean incision and was successfully managed using conservative treatment involving uterine cavity lavage.

## 2. Case Presentation

A 38-year-old woman (gravida 4, para 4) had vaginally delivered her first 2 children at another hospital. The third child, also born at another hospital, was delivered by cesarean section at 37-week gestation because of a low-lying placenta. The fourth child was born, at our hospital, at 37 weeks and 6 days of gestation via a cesarean section.

**Figure 1:**
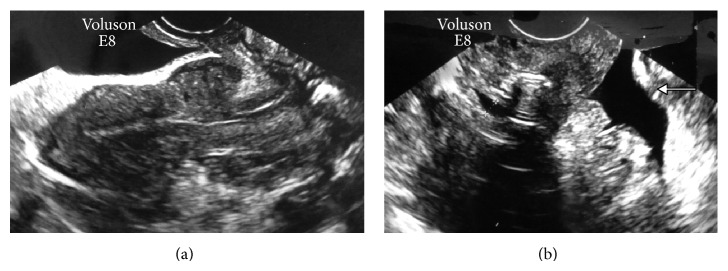
Ten months after the cesarean section. (a) Transvaginal ultrasonography shows a normal-sized uterus, but a clot-like mass with a low-to-iso-echoic inhomogeneous content, measuring 20 × 15 mm, is shown at a location corresponding to the site of a uterine incision in the vesicouterine pouch. (b) Saline infusion sonohysterography was performed using a physiological saline solution. During the saline infusion the uterine lumen did not expand and a fistula, measuring 4 mm in diameter, was observed (+- - -+). The images show that the physiological saline solution flowed out and spread from the uterine cavity into the peritoneal cavity through the vesicouterine pouch. In addition, the findings show that when the infusion continued, the saline solution accumulated in the Pouch of Douglas (arrow).

Nine months after the cesarean section, the patient's menstruation resumed; however, the amount of blood discharged was little, but persisted for one month. TVUS showed the presence of a normal-sized uterus with a 20 × 15-mm, blood clot-like mass in a vesicouterine pouch in the vicinity of the uterine incision from the previous cesarean section (Figure 1(a)). Saline infusion sonohysterography (SIS) was performed using a Hyscath hysterosalpingography catheter, and the findings showed that the uterine lumen did not dilate and that the physiological saline solution flowed from inside the uterine cavity into the peritoneal cavity through the vesicouterine pouch. As the infusion continued, the physiological saline solution accumulated in the Pouch of Douglas (Figure 1(b)). This led to a diagnosis of a menstrual fistula caused by the dehiscence of the cesarean section incision from the myometrium to the serosa. The process of infusing and recollecting the physiological saline solution (10 mL per time) was repeated 10 times with the Hyscath. Gradually, the recollected liquid changed from a dark red, viscous liquid to a light brown, serous liquid. Antibiotics (oral cephem; 300 mg/day for 5 days) were prescribed to prevent peritonitis. We referred to this procedure as lavage of the wound dehiscence.

We discussed treatment options with the patient, and she agreed to undergo a total hysterectomy, but not immediately because she was breastfeeding and rearing an infant. Therefore, we proposed attempting to treat her with a procedure with which we did not have any experience. The procedure consisted of regular lavages of the wound dehiscence to gradually remove the blood clots. By doing so, we expected the chronic inflammation at the site to improve and the myometrium to potentially heal spontaneously. The patient consented to the suggested therapy.

**Figure 2:**
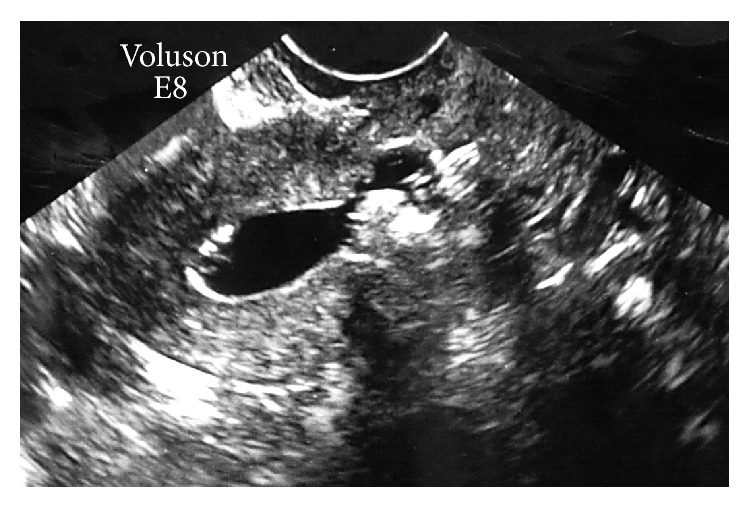
Eleven months after the cesarean section. When saline infusion sonohysterography was performed, physiological saline solution remained in the uterine cavity and did not flow into the vesicouterine pouch; expansion of the uterine lumen is evident.

The prolonged menstruation had stopped 5 days after the first lavage. Two weeks after the first lavage, a second lavage was conducted and the size of the blood clot had decreased to 15 × 13 mm; the uterine cavity also did not dilate due to the SIS. One week later (at the time of the third lavage), TVUS indicated that the blood clot had decreased to 12 × 10 mm. Two days after the third lavage, the patient began menstruating and demonstrated prolonged menstruation. Approximately 2 weeks after the third lavage, SIS was performed again (the fourth lavage). However, the saline did not flow into the vesicouterine pouch; instead, it remained in the uterine cavity, resulting in dilation of the uterine cavity. The blood clot in the vesicouterine pouch had spontaneously disappeared. Although a cesarean scar diverticulum “isthmocele,” measuring approximately 4 mm was found, spontaneous healing of the myometrium on the surface of the uterine serosa was confirmed (Figure 2). Over the course of the following month, SIS was performed 2 additional times. After, there was evidence that the spontaneous healing of the uterine incision persisted, with a muscle layer on the surface of the uterine serosa being clearly observed.

**Figure 3:**
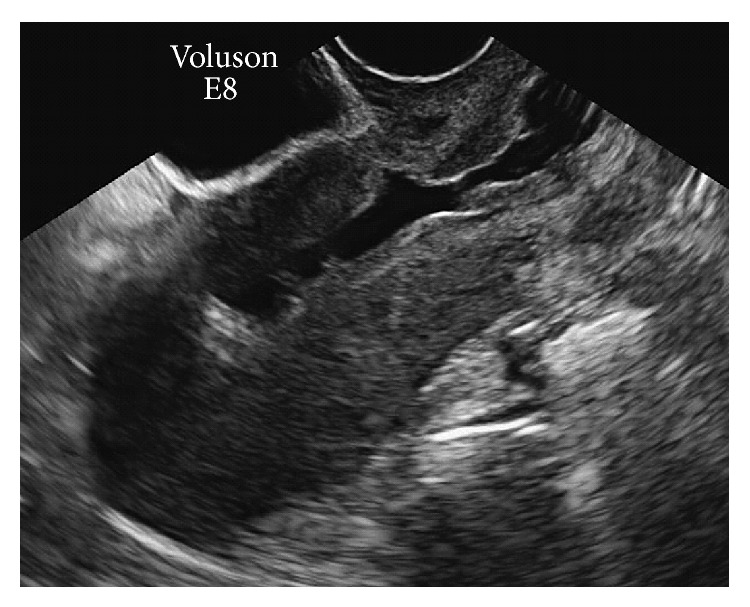
Nineteen months after the caesarean section. Saline infusion sonohysterography shows that despite the presence of a 4 mm defect “isthmocele,” myometrium continuity was maintained.

Finally, 19 months after the cesarean section, SIS was again performed (the seventh lavage) and a 4 mm defect “isthmocele,” was found; however, the healing of the muscle layer was maintained (Figure 3). Although the prolonged menstruation persisted, the amount of bleeding was extremely small, and the duration was shorter; therefore, the patient did not wish to undergo further treatment.

## 3. Discussion

The increase in the number of cesarean sections in recent years makes it easy to imagine that there has been an increase in the number of associated postoperative complications. Well-known complications include thromboembolisms, uterine ruptures, placenta accreta, placenta previa, and cesarean section incision pregnancies. However, recently, the muscle layer at the site of the uterine incision has been found to be thinner or absent in some cases and has been visualized as a CSD via TVUS. Since CSDs cause prolonged menstruation, irregular genital bleeding, and secondary infertility, the condition has attracted attention as a cesarean scar syndrome.

The incidence of CSD reportedly ranges between 6.6% and 69% [2–5], varying considerably depending on the author of the report. This is due to the absence of established standard diagnostic criteria for CSD. Among patients with a history of cesarean section and complaints of prolonged menstruation, defects have been reported in 82.6–100% of the cases [6, 7]. The availability of simple methods for diagnosing CSD would help to more firmly establish the true incidence of CSD. Among the methods for detecting CSD, TVUS and SIS are the simplest and most useful [8, 9]. In the TVUS, the CSD can be detected as a defect appearing as a triangular or dome-shaped echo-free space, which has been referred to by Monteagudo et al. as a “niche” [9]. In addition, Gubbini et al. [6] named it “isthmocele,” whereas for Regnard et al., a niche with a depth accounting for 80% or more of the muscle layer in the anterior wall of the uterus was referred to as “dehiscence” [10]. Performing SIS allows the TVUS results to be clearer and facilitates an accurate diagnosis. For that reason, patients with CSD symptoms, such as prolonged menstruations, should undergo SIS, in addition to TVUS.

In addition to providing a better estimate of the incidence of CSD, methods that facilitate the diagnosis of these defects may also help to minimize complications associated with the condition. For example, women with CSD-associated prolonged menstruation demonstrate some extent of menstrual blood accumulation in the “isthmocele.” As menstruation continues, the accumulated blood gradually flows out through the vagina. In cases of secondary infertility, the blood accumulation in the “isthmocele” may cause deterioration of the cervical mucus quality, block the passage of spermatozoa into the upper part of the uterus, and inhibit the implantation of fertilized eggs [5, 7].

The treatment of CSD in patients who desire to bear children requires the use of surgical repair. In the past, surgical repair required a laparotomy; recently, laparoscopic surgical repair has also been reported [11]. In women who do not desire to bear children, the treatment choices have involved either low doses of monophasic contraceptives or total hysterectomies. A total hysterectomy is a radical surgical treatment that many patients may be reluctant to undergo. There have also been recent reports of symptom improvement after resectoscopic surgery for removal of the flap-shaped fibrous tissue at the site of the scar [12, 13]. Based on the current report, a more conservative treatment seems to be possible.

In this study, we treated a CSD with physiological saline lavages, beginning 10 months after the patient's cesarean section. After 3 lavages, spread over approximately 1 month, the surface of the uterine serosa had healed, and the menstrual fistula had disappeared. An additional 4 lavages were performed, and spontaneous healing of the dehiscent muscle layer was successfully achieved, without any complications. Despite the favorable outcome, a number of issues remain unclear, including the reason lavage resulted in spontaneous healing (one possibility is that the chronic inflammation at the site of the dehiscence may have improved), the duration after cesarean section at which this procedure can be performed and remain effective, the types of defects this method is effective for, the appropriate amount of lavage fluid to be used for each lavage, and the number of times lavage should be performed before being considered ineffective, in cases where improvement does not occur. Answers to these questions will require additional experience with this technique.

In this study, we treated a patient with CSD, presenting as dehiscence of a cesarean incision site and prolonged menstruation by using physiological saline lavages at the site to achieve spontaneous healing. Additional cases will be needed in the future to determine the indications of this therapeutic procedure and to standardize the method. Because there has been no other report affirming the effectiveness of conservative treatment consisting of a lavage using a physiological saline solution as a treatment of CSD, this paper might be the first report on this issue.
